# Residual Disease in a Novel Xenograft Model of *RUNX1*-Mutated, Cytogenetically Normal Acute Myeloid Leukemia

**DOI:** 10.1371/journal.pone.0132375

**Published:** 2015-07-15

**Authors:** Umayal Sivagnanalingam, Marlene Balys, Allison Eberhardt, Nancy Wang, Jason R. Myers, John M. Ashton, Michael W. Becker, Laura M. Calvi, Jason H. Mendler

**Affiliations:** 1 Department of Medicine, James P. Wilmot Cancer Institute, University of Rochester Medical Center, Rochester, New York, United States of America; 2 Department of Pathology & Laboratory Medicine, University of Rochester Medical Center, Rochester, New York, United States of America; 3 Genomics Research Center, University of Rochester Medical Center, Rochester, New York, United States of America; 4 Department of Microbiology & Immunology, James P. Wilmot Cancer Institute, University of Rochester Medical Center, Rochester, New York, United States of America; University of Texas M.D. Anderson Cancer Center, UNITED STATES

## Abstract

Cytogenetically normal acute myeloid leukemia (CN-AML) patients harboring *RUNX1* mutations have a dismal prognosis with anthracycline/cytarabine-based chemotherapy. We aimed to develop an in vivo model of *RUNX1*-mutated, CN-AML in which the nature of residual disease in this molecular disease subset could be explored. We utilized a well-characterized patient-derived, *RUNX1*-mutated CN-AML line (CG-SH). Tail vein injection of CG-SH into NOD scid gamma mice led to leukemic engraftment in the bone marrow, spleen, and peripheral blood within 6 weeks. Treatment of leukemic mice with anthracycline/cytarabine-based chemotherapy resulted in clearance of disease from the spleen and peripheral blood, but persistence of disease in the bone marrow as assessed by flow cytometry and secondary transplantation. Whole exome sequencing of CG-SH revealed mutations in *ASXL1*, *CEBPA*, *GATA2*, and *SETBP1*, not previously reported. We conclude that CG-SH xenografts are a robust, reproducible in vivo model of CN-AML in which to explore mechanisms of chemotherapy resistance and novel therapeutic approaches.

## Introduction

Cytogenetically normal acute myeloid leukemia (CN-AML) patients harboring *RUNX1* mutations have a poor prognosis with standard chemotherapy [1–5]. In our previous study, not a single *RUNX1*-mutated, CN-AML patient experienced prolonged disease-free survival with anthracycline/cytarabine-based chemotherapy [2]. This suggests that residual disease is uniformly present in *RUNX1*-mutated, CN-AML patients treated with standard chemotherapy; yet the mechanisms underlying this remain poorly understood. To date, there exist no in vivo models of *RUNX1*-mutated CN-AML in which to define mechanisms underlying residual disease. Munker et al. generated a *RUNX1*-mutated, CN-AML cell line (CG-SH) from an AML patient and characterized its properties in vitro [6]; however, its potential to model the disease in vivo has not previously been explored. Moreover, CG-SH has not been comprehensively examined for additional AML-associated mutations that might contribute to its biological properties. Our hypothesis was that CG-SH cells would efficiently engraft immune-deficient mice and demonstrate residual disease after anthracycline/cytarabine-based chemotherapy, rendering it a robust, in vivo platform to study mechanisms of chemotherapy resistance and novel therapeutic approaches in CN-AML harboring *RUNX1* mutations.

## Materials and Methods

### Mice

NOD scid gamma (NSG) mice (Jackson Laboratories, Bar Harbor, ME, USA) were bred and maintained according to Institutional Animal Care and Use Committee (IACUC) policies at the University of Rochester Medical Center. All mouse experiments were conducted in accordance to institutional guidelines. Animals were monitored daily during treatment. To minimize suffering of mice during chemotherapy treatment, they were given HydroGel packs for better hydration and moist chow if they became lethargic and could not reach the food area of the cage. Mice were sacrificed by CO_2_ and cervical dislocation.

### Transplantation

The CG-SH cell-line [6] (provided by Dr. Reinhold Munker, Louisiana State University-Shreveport) was cultured in RPMI (Life Technologies, Grand Island, NY, USA)+12% fetal bovine serum (FBS; HyClone Laboratories, Logan, UT, USA)+penicillin/streptomycin. For transplantation studies, CG-SH cells were resuspended in 200 μl dPBS/0.5% FBS and injected via tail vein into non-irradiated NSG mice aged 8–10 weeks. To assess leukemia initiating ability of residual leukemic cells, non-irradiated secondary NSG recipients were transplanted with unsorted cell populations containing 5e4 CG-SH cells and engraftment by flow cytometry was determined 6 weeks later.

### Blood cell analysis

To assess complete blood counts, blood was obtained from individual mice and collected into EDTA-coated microtainer tubes (Beckton Dickinson, Franklin Lakes, NJ, USA). To determine white blood cell, platelet and hemoglobin levels, 20 μl blood was used for CBC-DIFF Veterinary Hematology System (HESKA) analysis.

### Tissue harvest

To harvest CG-SH cells from engrafted mice, peripheral blood, spleen and bone marrow were processed as follows. Peripheral blood was collected into EDTA-coated tubes and 500 μl 2% Dextran sulfate (Research Organics Inc., Cleveland, OH, USA) in dPBS was added to blood and was incubated for 20 minutes at 37°C. Post-incubation, peripheral blood mononuclear cell (PBMC)-enriched supernatant was collected, spun down and washed once with dPBS/0.5% FBS. Cells were red cell depleted by ammonium chloride lysis and washed with dPBS/0.5% FBS. Femurs were collected from mice, then flushed with dPBS/0.5% FBS (to obtain bone marrow samples) and spleens were collected and crushed through nylon mesh filters (to obtain spleen samples). Harvested cells were red cell depleted as previously described and washed with dPBS/0.5% FBS. After resuspension in dPBS/0.5% FBS, cells were filtered through polystyrene round-bottom tube with cell-strainer cap (Falcon, Corning, NY, USA) to create single-cell suspensions.

### Flow cytometric analysis

Harvested cells were stained with antibodies against mouse (clone 30-F11, BD Pharmingen, San Jose, CA, USA) and human (clone HI30, BD Pharmingen, San Jose, CA, USA) CD45. Flow cytometry was performed on an LSR II (BD Biosciences, San Jose, CA, USA) and data analyzed using FlowJo software (TreeStar, Ashland, OR). CG-SH engraftment levels were determined by quantifying the percentage of human CD45 positive (hCD45+) cells by flow cytometry.

### Limiting dilution analysis

CG-SH cells were serially diluted from 1e6 -1e1 and transplanted into non-irradiated NSG mice. For dilutions containing fewer than 1e6 cells, the total cell dose was brought to 1e6 prior to transplantation by adding syngeneic donor splenocytes. Engraftment was measured 10 weeks post-transplantation by flow cytometry.

### Chemotherapy treatment of CG-SH xenografts

Non-irradiated NSG mice were injected with 1e6 CG-SH cells via tail-vein. Five weeks after injection, mice were treated with 50 mg/kg/day of cytarabine (Mylan Institutional LLC., Schaumburg, IL, USA) for 5 days and 1.5 mg/kg/day of doxorubicin (APP Pharmaceuticals LLC., Rockford, IL, USA) for 3 days as recently reported [7]. On days 1–3, cytarabine and doxorubicin were co-administered via tail vein and on days 4 and 5, cytarabine was administered alone via intraperitoneal injection. Cytarabine and doxorubicin were diluted with bacteriostatic 0.9% NaCl (Hospira Inc., Lake Forest, IL, USA) prior to treatment. Chemotherapeutics were administered in a volume of 10 μl/gram of body weight. Weights were taken daily during treatment to ensure consistent dosing. The control group received saline alone at 10 μl/gram of body weight. All analyses of residual leukemic cells were performed 4 days after the completion of chemotherapy treatment.

### Bone marrow histology

To assess bone marrow histology, harvested hind limbs were fixed in 10% neutral-buffered formalin for at least 48 hours, decalcified in 14% EDTA, pH 7.2, for 10 days, and processed as previously described [8]. Histological sections (4 μm thickness) were stained with hematoxylin and eosin (H&E) to visualize overall morphology.

### Whole exome sequencing and analysis of sequence variations

Whole exome sequencing and identification of sequence variants was done in conjunction with the Genomics Research Center at the University of Rochester Medical Center. Whole exome sequencing data was analyzed for variations in 56 genes known to be recurrently mutated in myeloid malignancies, including 54 that are part of the TruSight Myeloid Sequencing Panel (illumina) plus *HNRNPK* and *FAM5C* [9]. Single nucleotide variations present in dbSNP and synonymous substitutions were not considered to be pathogenic. Mutations were confirmed by Sanger sequencing. See supporting information for further details (S1 Methods).

### Determination of cytogenetic and molecular evolution in engrafted mice

1e6 CG-SH cells from culture were engrafted into three different non-irradiated NSG mice. Six weeks after engraftment, CG-SH sorted from the bone marrow of each individual mouse, along with pre-engrafted CG-SH, were subjected to routine karyotyping and next generation sequencing analysis using the TruSight Myeloid Sequencing Panel (Illumina, San Diego, CA) per manufacturers’ recommendations. Sequence variations in engrafted CG-SH from each mouse were compared to those in pre-engrafted CG-SH to determine if new variations occurred after engraftment. See supporting information for further details (S1 Methods).

### Ethics Statement

Animals for this study were bred and maintained according to Institutional Animal Care and Use Committee (IACUC) policies at the University of Rochester Medical Center, which is NIH-assured, and Association for Assessment and Accreditation of Laboratory Care International-accredited. All protocols were approved by University Committee on Animal Resource (2005-256R) and Institutional Biosafety Committee (Becker-10-056).

### Statistics

Data are presented as mean+/-standard deviation (SD). Analysis was done by Student’s t-test using GraphPad Prism (v5.0).

## Results

### Engraftment Properties and Mutation Analysis of CG-SH Cells

**Fig 1:**
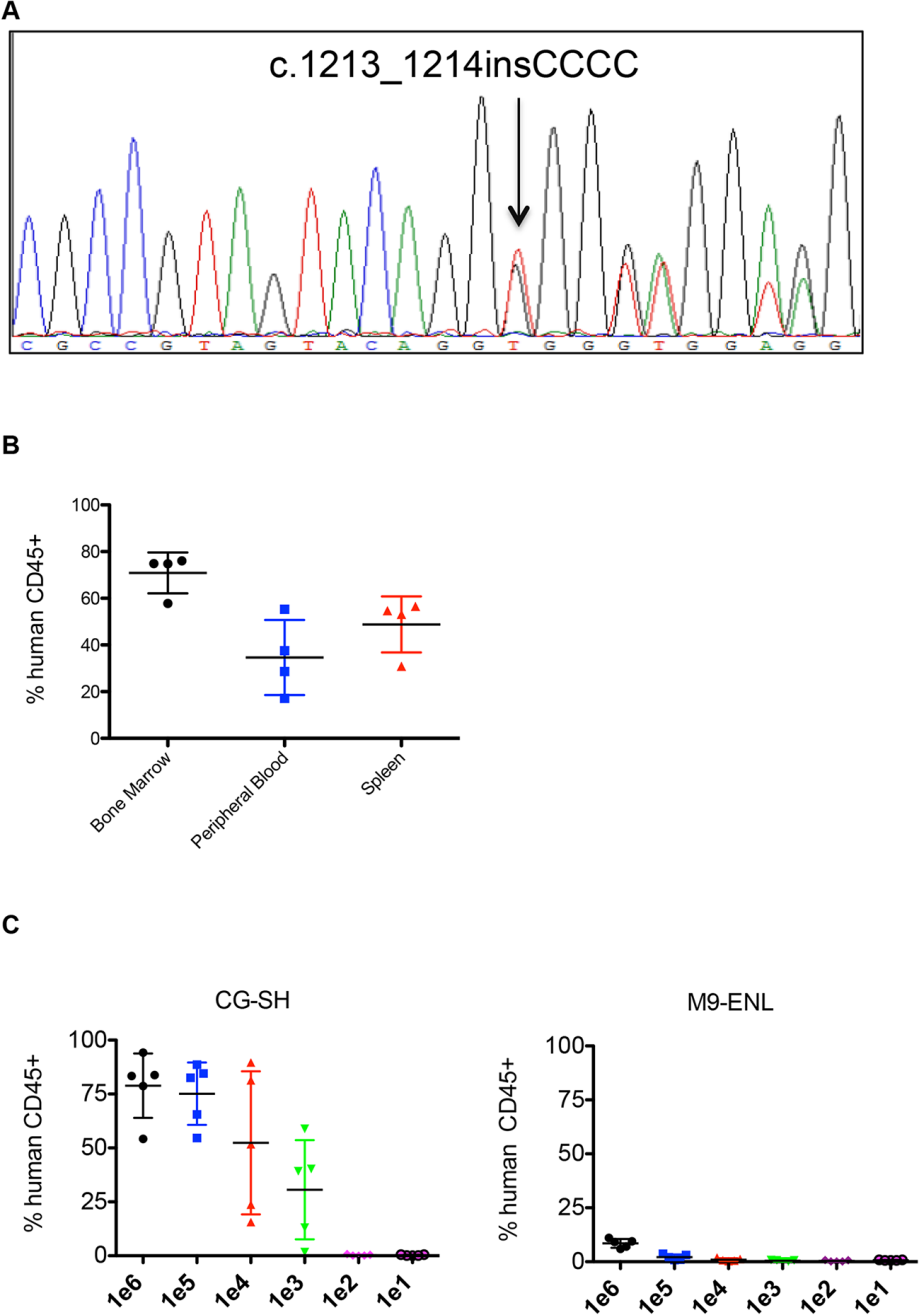
Engraftment Properties of CG-SH cells. **(A)** Sequencing chromatogram demonstrating the *RUNX1* mutation in CG-SH. **(B)** Percent hCD45+ engraftment achieved in the bone marrow (BM), peripheral blood (PB), and spleen of non-irradiated NSG mice receiving 1e6 CG-SH cells directly from cell culture via tail vein injection. Each symbol represents a single animal analyzed 6 weeks after transplantation. The horizontal black bars indicate mean engraftment. **(C)** Limiting dilution analysis of CG-SH and M9-ENL cells to determine the lowest cell dose necessary to achieve engraftment in each cell population. 1e1 – 1e6 cells were injected into non-irradiated NSG mice (5 mice per cell dose) and engraftment levels analyzed 10 weeks post- transplantation.

Initially, CG-SH cells were confirmed to harbor the previously reported mutation in exon 8 of *RUNX1* (c.1213_1214insCCCC; Fig 1A) and to be cytogenetically normal by routine karyotyping. Since our goal was to assess the response of CG-SH-engrafted NSG mice to AML-like chemotherapy, and mice pre-conditioned with irradiation do not tolerate this therapy, we first tested CG-SH engraftment in non-irradiated NSG mice. Tail vein injection of 1e6 CG-SH cells directly from culture into non-irradiated NSG mice resulted in high-level engraftment; at 6 weeks, mean engraftment in bone marrow, spleen, and peripheral blood was 71+/-9%, 49+/-12%, and 35+/-16%, respectively (Fig 1B). To determine the fewest number of CG-SH cells necessary to establish leukemia in NSG mice, we performed limiting dilution analysis. As few as 1e3 CG-SH cells were sufficient to establish leukemia in non-irradiated NSG mice (Fig 1C). At an equivalent cell dose, CG-SH engraftment levels were approximately 100-fold greater than those of M9-ENL cells, a different leukemia line [10] commonly used for engraftment studies in NSG mice (Fig 1C). To better define the spectrum of potential driver mutations in CG-SH, we conducted whole exome sequencing and analyzed genes recurrently mutated in myeloid malignancies [9,11]. In addition to the *RUNX1* and *NRAS* mutations previously reported, we identified mutations in *ASXL1*, which frequently co-occur with *RUNX1* mutations in CN-AML [2], *CEBPA*, *SETBP1*, and *GATA2* (Table 1). The *ASXL1* mutation, c.1900_1922del23, is a recurring structural abnormality in CN-AML [12]. All mutations were present at an allele frequency of 50%, suggesting that they are heterozygous and present within each cell. Given our interest in CG-SH as an in vivo model, we wished to determine if CG-SH cells demonstrate cytogenetic or molecular evolution after engraftment. CG-SH cells were isolated from the bone marrow of three individual NSG mice 6 weeks post-tail vein injection and subjected to cytogenetic and molecular analyses. Molecular analysis was conducted on pre- and post-engrafted CG-SH using a targeted next generation sequencing panel covering genes known to be involved in myeloid malignancies (Illumina). Relative to pre-engrafted CG-SH, engrafted CG-SH harbored no new cytogenetic or molecular abnormalities, suggesting that in vivo leukemia development in CG-SH xenografts is not accompanied by cytogenetic or molecular evolution.

**Table 1:** AML-associated mutations identified in CG-SH by whole exome sequencing.

*AML-associated mutations identified in CG-SH			
Gene	NCBI accession number	DNA sequence change	Deduced change in Protein Sequence
*RUNX1*	NM_001754	c.1213_1214insCCCC	p. L405Pfs*196
*ASXL1*	NM_015338	c.1900_1922del23	p.E635Rfs*15
*CEBPA*	NM_004364	c.572_573delinsT	p.P192Rfs*126
*NRAS*	NM_002524	c.37G>C	p.G13R
*SETBP1*	NM_001130110	c.681_682insTCTT	p.T228Sfs*8
*GATA2*	NM_001145661	c.1123G>C	p.L375V

*All mutations were present at an allele frequency of 50%

### Residual Disease in the Bone Marrow of CG-SH Xenografts Treated with Anthracycline/Cytarabine-based Chemotherapy

**Fig 2:**
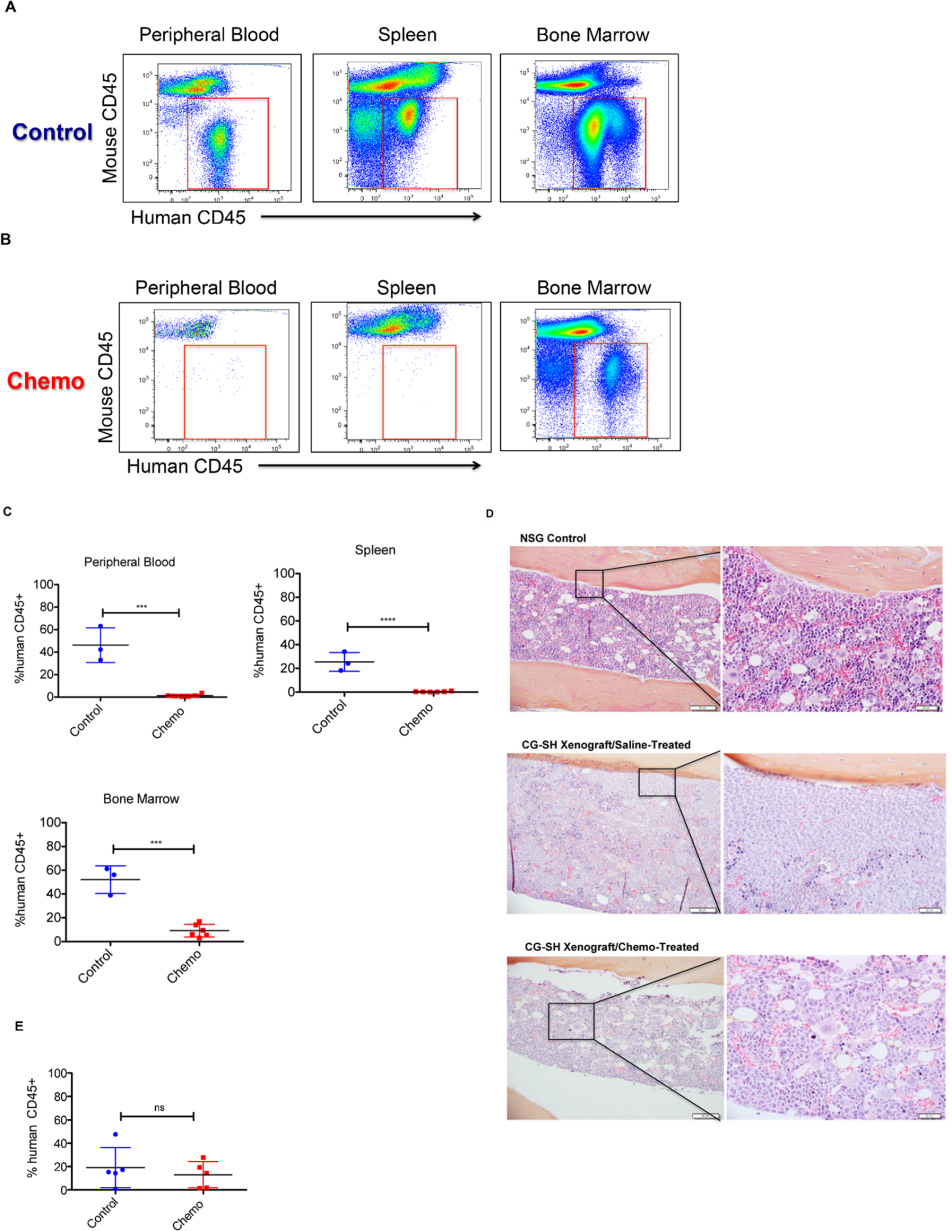
Residual Disease in CG-SH Xenografts Treated with a 5-day regimen of Anthracycline/Cytarabine-Based Chemotherapy. **A and B.** Flow cytometric plots demonstrating the level of CG-SH engraftment in the bone marrow, peripheral blood, and spleen of a representative saline-treated control mouse **(A)** and a representative chemotherapy-treated mouse **(B)**. Treatment was initiated five weeks after tail vein injection of 1e6 CG-SH cells into non-irradiated NSG mice. Mice were analyzed four days after the completion of chemotherapy. **(C)** Quantification of the impact of chemotherapy in a cohort of CG-SH- xenografted mice. Each symbol represents a single mouse analyzed four days after the completion of treatment. Control mice were treated with saline. **(D)** Bone marrow histology four days after the completion of chemotherapy in CG-SH xenografts. Magnification is at 20x (left panels) and 40x (right panels), respectively. **(E)** Chemotherapy fails to eradicate leukemia-initiating cells in CG-SH xenografts. Leukemic cells were harvested from the bone marrow of chemotherapy-treated CG-SH xenografts or saline-treated controls and tested for their ability to engraft secondary NSG recipients. Non-irradiated secondary recipients were transplanted with unsorted cell populations containing 5e4 CG-SH cells and engraftment was determined 6 weeks later. All data were analyzed by the comparison of means using unpaired t-test. ***p = 0.0001, ****p<0.0001, ns = not significant.

Given the chemotherapy resistance demonstrated by patients with *RUNX1*-mutated, CN-AML, we hypothesized that CG-SH xenografts would demonstrate residual disease after anthracycline/cytarabine-based chemotherapy. To test this idea, CG-SH xenografts were treated with a 5-day regimen of doxorubicin and cytarabine [7]. Disease response was assessed 4 days after completion of chemotherapy. As expected, mice xenografted with CG-SH developed leukemia in the bone marrow, peripheral blood, and spleen (Fig 2A and 2C). Chemotherapy treatment resulted in clearance of leukemic cells from the peripheral blood and spleen by 4 days after the completion of treatment (Fig 2B and 2C). In contrast, chemotherapy failed to clear leukemic cells from the bone marrow at the same point in time (Fig 2B, 2C and 2D) although it caused significant pancytopenia in treated mice (S1 Fig). We next wanted to know if residual leukemic cells were capable of re-establishing a high leukemic burden post-treatment; however, this analysis was precluded by the fact that chemotherapy-treated CG-SH xenografts were very ill and died within 1–2 weeks of completing therapy. Although a formal survival analysis was not performed, saline-treated CG-SH xenografts were generally much healthier and longer-lived than their chemotherapy-treated counterparts. It has been hypothesized that the reason why chemotherapy fails to cure most AML patients is due to inadequate targeting of leukemic populations capable of initiating and maintaining the disease. To determine if chemotherapy fails to eradicate leukemia initiating cells within CG-SH xenografts, we tested the ability of residual bone marrow CG-SH cells to initiate leukemia in secondary transplants. As demonstrated in Fig 2E leukemic cells from chemotherapy-treated animals were able to engraft secondary recipients to a similar degree as those from saline-treated controls. Thus, anthracycline/cytarabine-based chemotherapy fails to eradicate leukemia-initiating cells from the bone marrow of CG-SH xenografts.

## Discussion

It is critical to develop in vivo models of high-risk cytogenetic and molecular subgroups of AML to define mechanisms of chemotherapy resistance and identify novel therapeutic approaches. In this study, we developed a robust, in vivo model of *RUNX1*-mutated CN-AML, a molecular subgroup of CN-AML that is relatively common, responds poorly to standard chemotherapy, and for which no molecularly targeted therapies exist. To our knowledge, this is the first report of an in vivo model of CN-AML, the most common cytogenetic subgroup of the disease, occurring in approximately 50% of AML patients. Because it cytogenetically mirrors the majority of human AML cases (unlike other available AML cell lines), we suspect that it will perform better than other lines in elucidating clinically relevant disease mechanisms. This model has several important strengths to enhance mechanistic and therapeutic analyses, including engraftment without conditioning irradiation, relatively short latency to leukemia development, and high disease burden in the bone marrow, spleen, and peripheral blood. Thus, it is an ideal platform to investigate leukemia/bone marrow niche interactions (without the confounding effects of radiation), to preclinically evaluate novel therapeutics, and to determine how different in vivo environments affect leukemic cell properties.

In almost all patients with *RUNX1*-mutated CN-AML, other AML-associated mutations co-exist. Thus, the extent to which the *RUNX1* mutation drives the disease, relative to the other co-existing mutations is unknown. Similar to patients, the *RUNX1* mutation does not occur alone within CG-SH; rather, five other AML-associated mutations are also present. Thus, we expect that CG-SH will be useful to determine the relative contribution of each co-existing mutation to key leukemic properties, such as leukemia initiation, differentiation block, and chemoresistance. Such results are expected to shed light on which mutations are most critical to therapeutically target when multiple are present within the same leukemic cell.

Chemotherapy response in our model mimics what is seen in most patients with this disease; leukemic cells were cleared from the periphery but not the bone marrow, offering the opportunity to identify both cell-intrinsic and bone marrow microenvironmental factors that protect residual AML cells from chemotherapy. Available data supports the idea that bone marrow microenvironmental protection is mediated by cross talk between bone marrow stromal cell and leukemic populations that results in quiescence and/or enhanced anti-apoptotic signaling in leukemic cells [13–18]; however, these mechanisms are incompletely understood, particularly as they pertain to specific molecular and cytogenetic subsets of AML. Further work should be directed toward better understanding mechanisms promoting residual disease in AML, ideally using genetically defined, in vivo models that faithfully recapitulate the disease-subgroup of interest. Definition of these mechanisms will result in novel approaches to the eradication of residual disease in AML.

## Supporting Information

S1 Methods (DOCX)

S1 Fig Peripheral Blood Counts in CG-SH Xenografts Treated with Chemotherapy. Peripheral blood counts four days after the completion of chemotherapy in CG-SH xenografts. White blood cell, platelet, and hemoglobin levels were measured using HESKA. “NSG controls” are non-engrafted mice not receiving chemotherapy.
(TIF)
