# Supplementary material for: Residual Disease in a Novel Xenograft Model of RUNX1-Mutated, Cytogenetically Normal Acute Myeloid Leukemia
Source: PLoS One. 2015 Jul 15;10(7):e0132375. doi: 10.1371/journal.pone.0132375 (PMC4503761; doi:10.1371/journal.pone.0132375)
Supplement: S1 Methods — (DOCX) [file pone.0132375.s001.docx]

**S1 Methods**

**Whole exome sequencing and analysis of sequence variations**

Purified genomic DNA was quantified using the Qubit Fluorometer (ThermoFisher, Waltham, MA) and quality was assessed with the gDNA tapestation assay (Agilent Technologies, Santa Clara, CA). (Whole exome libraries were generated using SureSelectXT Whole Exome v5 (+UTRs) per manufacturer’s recommendations (Agilent Technologies, Santa Clara, CA). Briefly, 4ug of good quality genomic DNA was sheared with Covaris S2 (Covaris, Woburn, MA) to an average peak size of 250bp followed by end repair, polyadenylation, Illumina adaptor ligation, and purification with AmpureXP (Beckman Coulter, Brea, CA). Library amplification was carried out with 250ng of purified DNA with 4 cycles of PCR followed by AmpureXP purification (Beckman Coulter, Brea, CA). Exome probe hybridization capture was performed per manufacturer’s protocols. Exome target enriched samples were quantified using the Qubit Fluorometer (ThermoFisher, Waltham, MA) and library sizing was assessed with the high sensitivity Bioanalyzer 2100 DNA assay (Agilent Technologies, Santa Clara, CA). Whole exome libraries were normalized, pooled and 2x100 pair end sequencing was performed with the HiSeq2500 (Illumina, San Diego, CA). Raw read data generated from the HiSeq2500 sequencer were demultiplexed using configurebcl2fastq.pl version 1.8.4. Quality filtering and adapter removal was performed using Trimmomatic version 0.32 with the following parameters: "SLIDINGWINDOW:4:20 TRAILING:13 LEADING:13 ILLUMINACLIP:adapters.fasta:2:30:10 MINLEN:25". Processed/cleaned reads were then aligned to the human genome reference hg19 with SHRiMP version 2.2.3. Variant analysis was performed using SAMtools.

**Determination of cytogenetic and molecular evolution in engrafted mice**

The target oligo pool was hybridized with 100ng of high quality genomic DNA. Extension and ligation of bound target oligos as performed followed by PCR amplification and PCR cleanup by AmpureXP purification (Beckman Coulter, Brea, CA). Library quantification was performed using Bioanalyzer 2100 (Agilent Technologies, Santa Clara, CA), Qubit Flourometer (ThermoFisher, Waltham, MA), and KAPA qPCR (Kapa Biosystems, Wilmington, MA) and subsequent DNA sequencer performed with MiSeq v2 2x150 (Illumina, San Diego, CA ). Variant analysis was performed using samtools version 1.0 "mpileup -q 5 -ugf " and bcftools version 1.0 "call -vmO v ". Annotation of the resulting SNP/INDEL calls was performed using Annovar (version date 2014-07-22) "table_annovar.pl --vcfinput --buildver hg19 --remove --protocol refGene,clinvar20140929,snp138 --operation g,f,f --nastring ."
