# Supplementary figures and images for: Residual Disease in a Novel Xenograft Model of RUNX1-Mutated, Cytogenetically Normal Acute Myeloid Leukemia
Source: PLoS One. 2015 Jul 15;10(7):e0132375. doi: 10.1371/journal.pone.0132375 (PMC4503761; doi:10.1371/journal.pone.0132375)

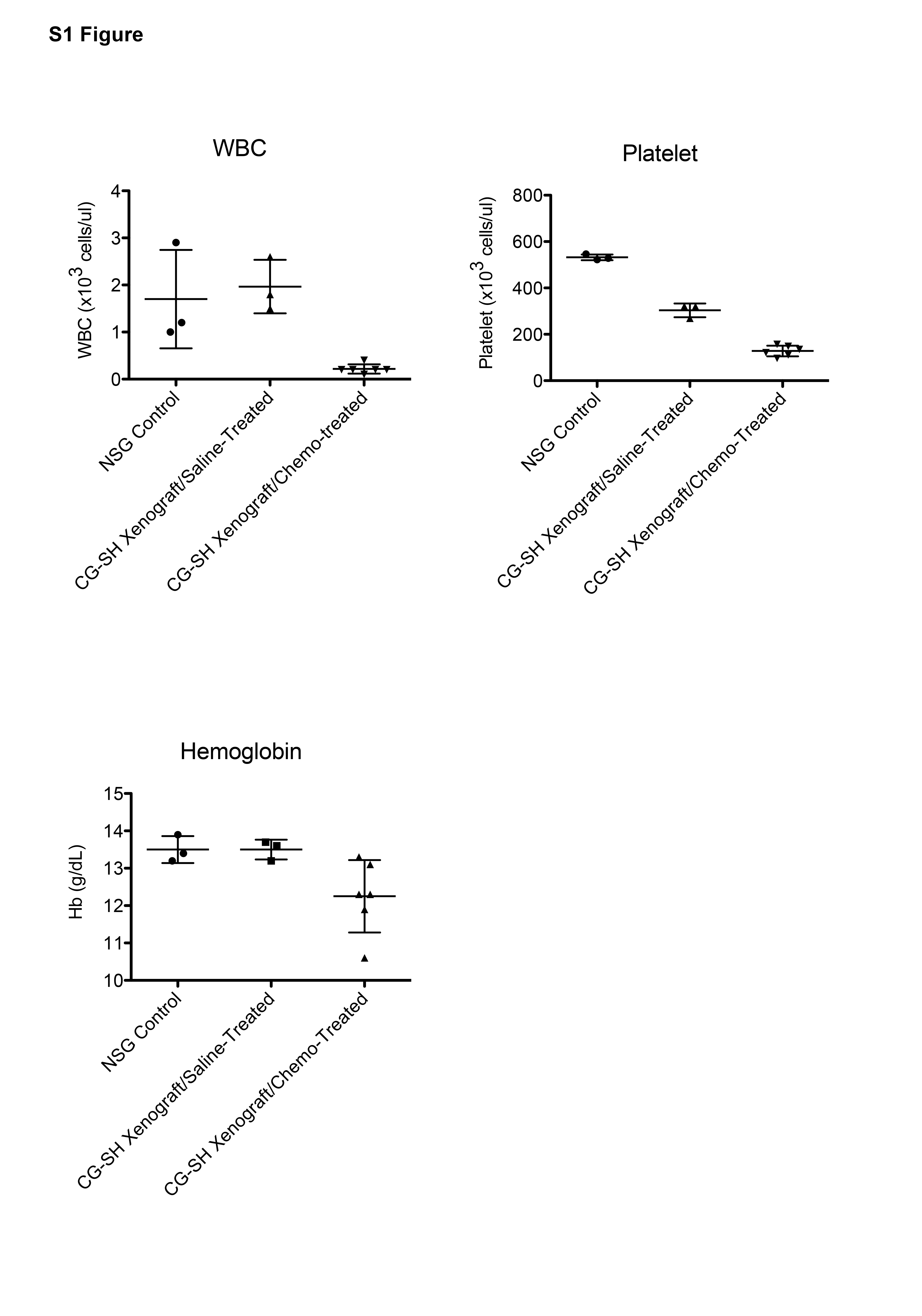

Supplement: S1 Fig — Peripheral blood counts four days after the completion of chemotherapy in CG-SH xenografts. White blood cell, platelet, and hemoglobin levels were measured using HESKA. “NSG controls” are non-engrafted mice not receiving chemotherapy. (TIF) [file pone.0132375.s002.tif]
